# Alternative Treatments for Indoor Residual Spraying for Malaria Control in a Village with Pyrethroid- and DDT-Resistant Vectors in The Gambia

**DOI:** 10.1371/journal.pone.0074351

**Published:** 2013-09-13

**Authors:** Julie-Anne A. Tangena, Majidah Adiamoh, Umberto D’Alessandro, Lamin Jarju, Musa Jawara, David Jeffries, Naiela Malik, Davis Nwakanma, Harparkash Kaur, Willem Takken, Steve W. Lindsay, Margaret Pinder

**Affiliations:** 1 Laboratory of Entomology, Wageningen University and Research Centre, EH Wageningen, Netherlands,; 2 Institut Pasteur du Laos, Vientiane, Lao PDR; 3 Disease Control and Elimination Theme, Medical Research Council Unit, Banjul, Gambia; 4 Statistics, Medical Research Council Unit, Banjul, Gambia; 5 Unit of Malariology, Institute of Tropical Medicine, Antwerp, Belgium; 6 National Malaria Control Programme, Kanifing, Gambia; 7 London School of Hygiene and Tropical Medicine, London, United Kingdom; 8 School of Biological and Biomedical Sciences, Durham University, Durham, United Kingdom; University of Crete, Greece

## Abstract

**Background:**

Malaria vector control is threatened by resistance to pyrethroids, the only class of insecticides used for treating bed nets. The second major vector control method is indoor residual spraying with pyrethroids or the organochloride DDT. However, resistance to pyrethroids frequently confers resistance to DDT. Therefore, alternative insecticides are urgently needed.

**Methodology/Principal Findings:**

Insecticide resistance and the efficacy of indoor residual spraying with different insecticides was determined in a Gambian village. Resistance of local vectors to pyrethroids and DDT was high (31% and 46% mortality, respectively) while resistance to bendiocarb and pirimiphos methyl was low (88% and 100% mortality, respectively). The vectors were predominantly *Anopheles* *gambiae* *s.s.* with 94% of them having the putative resistant genotype *kdr* 1014F. Four groups of eight residential compounds were each sprayed with either (1) bendiocarb, a carbamate, (2) DDT, an organochlorine, (3) microencapsulated pirimiphos methyl, an organophosphate, or (4) left unsprayed. All insecticides tested showed high residual activity up to five months after application. Mosquito house entry, estimated by light traps, was similar in all houses with metal roofs, but was significantly less in IRS houses with thatched roofs (p=0.02). Residents participating in focus group discussions indicated that IRS was considered a necessary nuisance and also may decrease the use of long-lasting insecticidal nets.

**Conclusion/Significance:**

Bendiocarb and microencapsulated pirimiphos methyl are viable alternatives for indoor residual spraying where resistance to pyrethroids and DDT is high and may assist in the management of pyrethroid resistance.

## Introduction

Vector control with long-lasting insecticidal nets (LLINs) and indoor residual spraying (IRS) has contributed greatly to the recent dramatic decline in malaria in sub-Saharan Africa [1]. For example, during the past decade vector control activities have been scaled up in The Gambia, with both LLIN and IRS with dichlorodiphenyltrichloroethane (DDT). These measures, together with increased availability of effective treatment, have been associated with a dramatic decline in malaria cases, prompting the view that elimination may be feasible [2]. The future, however, is threatened by the increasing resistance of the vectors to the pyrethroids used for LLINs and IRS [3,4]. Vector resistance to both pyrethroids and DDT have been reported in many parts of Africa, including West Africa, including Côte d’Ivoire and Burkina Faso [3,5,6]. Since cross resistance could compromise the effectiveness of both IRS and LLINs programmes, it is essential to monitor it and evaluate alternative insecticides for IRS [4].

There are only two alternative classes of insecticides currently available for malaria control, namely carbamates and organophosphates. The World Health Organisation’s Pesticide Evaluation Scheme approves several insecticides in these classes for IRS including bendiocarb, a carbamate, and pirimiphos methyl, an organophosphate [7,8]. Their mode of action differs from that of pyrethroids and DDT, they prevent acetylcholine breakdown [9], so they could be used in combination or rotation for the management of insecticide resistance where pyrethroid-treated LLINs are used [10].

Bendiocarb, at the recommended concentration, has an estimated residual activity of two to seven months [8,10]. Although reports on pirimiphos methyl residual activity vary from two to three months [8,11,12], it has been recently formulated in microcapsules which reduces the persistent unpleasant odour associated with its use and may prolong its residual activity.

Vector (*Anopheles gambiae* s.l.) resistance to both carbamates and organophosphates has been reported in Côte d’Ivoire and Burkina Faso [13,14] and may have spread. Neither bendiocarb nor pirimiphos methyl, however, had been previously used for IRS in The Gambia, nor in neighbouring Senegal. The current study compared the efficacy and acceptability of bendiocarb, microencapsulated pirimiphos methyl and DDT in a Gambian village and measured insecticide susceptibility of the vectors.

## Materials and Methods

### Study site

The study was carried out in Sare Alpha village (13°21' 38 N, 13°58' 50 W), in the Upper River Region of The Gambia, an area of open Sudanian savannah. There is a single rainy season (June to October) with malaria transmission mainly from August to December. The villagers live in delineated compounds with an average of 28 people and 10 dwelling rooms. Most houses are made of mud or cement with interior walls cemented and matt painted (67%) or left bare. Roofs are either thatch (51%) or metal and nearly every house has closed eaves (93%). The village received LLIN from the government countrywide distribution in March and June 2011 and the project provided additional LLIN (Olyset® Nets, Sumitomo Chemical Company, Japan) in July 2011 to achieve 97% (1754/1808 sleeping places) coverage.

### Insecticide application

The 104 residential compounds were stratified into large (those with 11-30 rooms) and small (5 to 10 rooms) and random sampling was used to select 16 compounds in each strata. Eight compounds were then selected randomly, four in each strata, for spraying with one of the three insecticides or left untreated. Insecticides were applied at concentrations recommended by WHO [8]: 0.4g/m^2^ bendiocarb (Ficam W, Bayer (Pty.) Ltd., Nigel, South Africa), 2g/m^2^ DDT (DDT 75% WP, Hindustan Insecticides Ltd, New Delhi, India) and 1g/m^2^ pirimiphos methyl (Actellic 300 CS, Syngenta, Seneffe, Belgium). IRS was conducted, 6-8^th^ July 2011, using Hudson X-pert sprayers at a rate of five seconds per 2m wall, by experienced spray-men from the Gambian National Malaria Control Programme under supervision and according to WHO guidelines [8].

During IRS, insecticidal sprays were sampled in 9-10 houses for each insecticide on Whatman No. 4 filter papers and the insecticide concentration was estimated by High Performance Liquid Chromatography, using Thermo, Fisher Scientific (Hemel Hempstead, UK) equipment and Chromeleon Version 6.80 SR11 software [15]. The results were expressed as grams of active ingredient /m^2^ by reference to calibration curves of each insecticide.

### Insecticide susceptibility testing

The susceptibility of wild caught *An. gambiae* s.l. was assessed using tube tests, viz. WHO bioassays [7]. Immature stages were collected from August to November 2011 in Sare Alpha and neighbouring Sare Juldeh (13°19' 32 N, 14°3' 36 W), transported to the insectary and reared to adults. Exposures were conducted at 29 ± 4°C and 63% relative humidity (range 45-75%). Approximately ten, three to five day old adult female mosquitoes were placed in holding tubes for 30 minutes and damaged mosquitoes removed. Remaining mosquitoes were then exposed for 60 minutes to insecticide-impregnated or appropriate control papers: (1) 0.1% bendiocarb, batch BE63; (2) 4% DDT, batch DD117; (3) 0.75% permethrin, batch PE190; (Vector Control Research Unit, University Sains Malaysia, Penang, Malaysia) (4) 0.025% pirimiphos methyl (Syngenta, Seneffe, Belgium). Insects were returned to the holding tube, given 10% glucose solution and mortality recorded after 24 hours. Live mosquitoes were those able to fly and any knocked down mosquito that had lost legs or wings was considered dead. Members of the *An. gambiae* complex were identified to species, M and S forms and *kdr* resistant markers by PCR [16,17,18]. 

### Assessment of insecticide residual activity

Persistence of insecticides on walls was measured using WHO cone tests [7] in four houses with bare-mud walls and four with matt-painted cement for each treatment arm; four unsprayed houses with mud walls served as controls. A breeding stock of insecticide susceptible *An. gambiae s.s*. M form, originating from Yaoundé, Cameroon was obtained in June 2011 from the Pasteur Institute in Dakar and maintained in an insectary at 27 ± 2°C, 70-80% relative humidity and a 12: 12 h light : dark photoperiod. Adult mosquitoes were maintained with 10% glucose solution *ad libitum*. Larvae were fed daily with Tetramin^®^ Fish Flakes (Melle, Germany). Adults from the colony, 20 mosquitoes/tube test, were tested for sensitivity to permethrin, deltamethrin and DDT in July 2010 and were 100% sensitive.

Tests were conducted two months post-IRS and then at monthly intervals for a further three times. Cages of three to five day old, female mosquitoes were taken to the field and transferred to the cones in batches of 20, exposed to walls for 30 minutes and knock down was recorded. Cones were attached to walls furthest from the door, to minimize risk of abrasion, at 70cm below the roof, 70cm above the floor and in the middle. The positions were marked and repeated were measurements made at the same place. Mosquitoes were then transferred into holding cups in a cool box, taken to the insectary and mortality recorded 24h post-exposure. The average temperature during exposures was 32°C (range 27-37°C) and relative humidity decreased with time: 75% (range 68-85%) in the second and third month post-IRS, 61% (range 19-75%) for the fourth month and 12% (range 2-25%) in the fifth month.

### Indoor sampling of mosquitoes

Eight sentinel rooms, in 32 separate residential compounds where a consenting adult slept under a bed net, were sampled for each intervention and the control arm using CDC light traps. Traps were positioned once a month, from 2^nd^ August to 2^nd^ December 2011, at the foot of the bed with the light 1m above the floor and operated throughout the night. Potential risk factors known to affect mosquito densities in The Gambia [19] were recorded at each capture. Specimens were frozen before morphological identification using established keys [20,21]. Members of the *An. gambiae* complex were identified to species, M and S forms, and *kdr* resistant markers by PCR as indicated above.

### Focus Group Discussions

Given the invasive nature of IRS, its possible effect on bednet acceptance and usage, the different odours of the three insecticides and odour duration, we set out to explore the perceptions, attitudes and practices of adult residents of IRS-houses using Focus Group Discussions (FGDs) in Sare Alpha five months post-IRS. The FDG guide was prepared collectively by the principle co-authors and centred on the impact of the insecticides on mosquito abundance, house appearance, net use and the acceptability of the spray process. Three FGDs of approximately seven participants were held for each insecticide; a male group, a younger female and an older female group (31 years or older). A trained moderator with experience in qualitative research methods whose first language was Fula, the dominant language of the village, and who was familiar with local dialects, customs and values helped the groups discuss using the pre-prepared topic guide following a standard method [22]. With the consent of the participants the discussions were tape-recorded, and later transcribed into Fula, translated into English and typed into a word package.

### Statistical analysis

Data were double-entered into Access databases, verified and validated by consistency checks. The analysis of the susceptibility tests followed WHO recommendations [7]. Fishers exact test was used to compare the susceptibility of mosquitoes to insecticides, the effect of the interventions on the presence of *An. gambiae* s.l. in light traps and to identify confounders. The Clopper-Pearson method was used to calculate 95% confidence intervals for proportions, unless 97.5% is stated. General Estimating Equations (GEE) population-averaged models with exchangeable correlation structure were fitted to ranked mosquito mortality from the cone-tests to compare insecticide residual activity and the influence of confounders. Random-effects logistic regression models were used to compare the presence or absence of mosquitoes in light traps by intervention arm and the influence of confounders on this. Statistical analysis used STATA (ver. 12, College Station, Texas, USA). FGDs were examined by content analysis using the pre-defined topics to create a code list and matching respondents comments to these, first within each FGD (Table S2) and then by insecticide. Where comments were congruent across the treatment arms these were presented together and in case of differences these were explored separately.

### Ethics statement

The study proposal was reviewed and approved by the Scientific Coordinating Committee of the Medical Research Council Unit, The Gambia, and subsequently ethical approval was granted by the Gambian Government / Medical Research Council Unit, Joint Ethics Committee and the London School of Hygiene and Tropical Medicine Ethics Committee. Verbal, informed consent was provided by the village leaders following information meetings and documented, verbal, informed consent was provided by household heads for compounds enrolled into the study. Room owners provided written, informed consent for the mosquito collections and the cone tests.

## Results

### Insecticide susceptibility

258 female *An. gambiae* s.l., raised from wild-caught larval specimens, were exposed to insecticides in tube tests at an average of 9.9 mosquitoes / tube. No mosquito (0/56; CI 0-6) died in the control tubes during the tests. The susceptibility of mosquitoes from Sare Alpha and neighbouring Sare Juldeh to DDT were similar (22/44 and 11/28, p=0.469) so the results were combined. Mortality 24h post-exposure was 46% (33/72; CI 34-58%) for DDT, 31% (16/52; CI 19-44%) for permethrin, 88% (37/42; CI 75-96) for bendiocarb, and 100% (26/26; 97.5% CI 87-100) for pirimiphos methyl.

More than half (58%, 148/257) of the female *An. gambiae* s.l. were *An. gambiae s.s.* and the rest were *An. arabiensis* (42%, 109/257); 90% (133/147) of the *An. gambiae* s.l. were S-form. *Kdr* 1014 genotypes were similar between villages but varied by species (Table 1). The 1014S (serine) mutation was only present in *An. arabiensis*, and at low frequencies, whilst the 1014F (phenylalanine) was present in >95% of *An. gambiae s.s*. S form but only in <20% of *An. arabiensis* (Table 1).

**Table 1:** Kdr 1014 genotypes present in wild larval caught *An*. *gambiae* s.l. by subspecies.

		**Codon 1014 Genotypes**						**Phenylalanine allele**	**Serine allele**
**Species**	**Village**	**LL**	**LS**	**LF**	**SF**	**FF**	**SS**	**% ± 95%CI**	**% ± 95%CI**
*An. arabiensis*	Sare Juldeh	29	4	2	0	7	2	18.0 (10.7-28.4)	9.1 (4.0-17.1)
	Sare Alpha	37	13	6	0	4	2	11.3 (6.3-18.1)	13.7 (8.2-21.0)
*An. gambiae* M	Sare Juldeh	3	0	0	0	5	0	62.5 (35.4-84.8)	0
	Sare Alpha	2	0	1	0	1	0	37.5 (8.5-75.5)	0
*An. gambiae S*	Sare Juldeh	1	0	1	0	29	0	95.2 (86.5-98.9)	0
	Sare Alpha	2	0	2	0	98	0	97.1 (93.7-98.9)	0

### Insecticide residual activity

The estimates for concentrations of insecticide sprayed were higher than expected for bendiocarb (target dose= 0.4g/m^2^, sprayed mean dose= 0.98g/m^2^, CI 0.83-1.12) and DDT (target dose= 2g/m^2^, sprayed mean dose= 3.44g/m^2^, CI 1.25-8.92), but within the expected range for pirimiphos methyl (target dose= 1g/m^2^, sprayed mean dose= 1.12 g/m^2^, CI 1.03-1.21).

**Figure 1:**
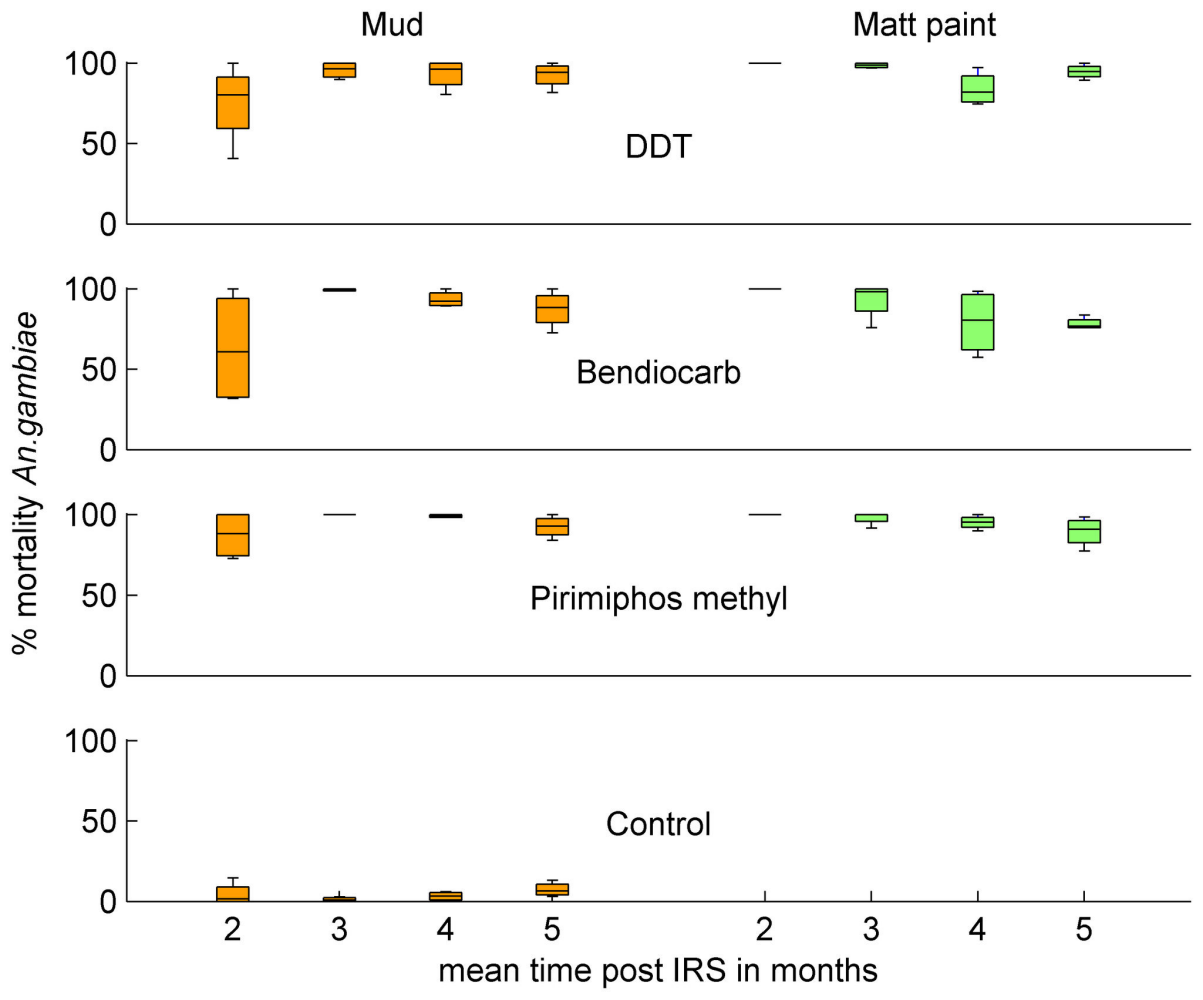
Insecticide residual activity during the first five months after IRS by insecticide.

Mortality in control cone tests did not vary significantly between cone position nor with time after IRS (median 3.02%, inter quartile range = 5.60, p=0.07 from linear regression on log transformed percentages). At the first sampling time, two months post-IRS, residual activity varied with wall surface being 100% on walls with matt paint and lower on mud walls (Figure 1). Tests conducted three months post IRS showed similar values for the two surfaces and by the fourth and fifth months there was a tendency for residual activity to be lower on walls with matt paint. GEE population-averaged models of mosquito mortality averaged across the three wall positions (Table S1) showed significant interaction between the wall surface and time after spraying (p<0.001) but there was no significant difference between the insecticides. Similar results were found using mortality from the three wall positions.

### Sampling of adult mosquitoes

*An. gambiae* s.l. females were collected in 33% of traps; 36% in the control rooms (14/39), and 30% in the IRS rooms (36/117). Most *An. gambiae* s.l. (98%, 128/130) were identified to subspecies; 41% were *An. arabiensis* and 59% *An. gambiae s.s.*, with the S form predominating in the latter (75%, 56/75). *Kdr* 1014 genotypes were identified for 53 *An. arabiensis* and 74 *An. gambiae s.s*. The results were similar to those of the larval caught specimens; the 1014S (serine) mutation was only present in *An. arabiensis* (gene frequency 20%, 21/106, CI 13-29%) and the 1014F (phenylalanine) predominated in *An. gambiae* s.s. (74%, 109/148, CI 66-81%), with only 13% in *An. arabiensis* (13/106, CI 7-20%).

The impact of IRS on presence of female members of the *An. gambiae* complex in traps was examined in a two-way table, but the proportions of traps with *An gambiae* s.l. were not significantly different in the presence or absence of IRS either when the catches from interventions were compared individually to control or when they were combined (Fishers exact test). However, several factors were significantly associated with presence of *An. gambiae* females in rooms: roofing material (metal or thatch), the interior wall surface (bare cement, bare mud or matt paint) and the number of people sleeping in the room. A formal multivariable analysis was performed using logistic regression with compound as a random-effect to examine these possible confounders and their interactions, together with those of open or closed eaves and the presence of a tethered horse. There was a significant interaction between roofing material and the different insecticides. Table 2 shows the odds ratios for IRS compared to control for metal and thatched roofs for each insecticide and adjusting for the number of people sleeping in the room. The effect was only significant for DDT in the presence of thatched roofs (Table 2), although a similar trend was apparent for all three insecticides

**Table 2:** The impact of IRS on female *An*. *gambiae* s.l. entering houses by roof type and insecticide.

			Logistic Regression ^*^		
**Insecticide**	**Roof**	***Angambiae* s.l. present / total traps (%**)	**OR**	**CI 95%**	**P**
Control	Metal	6/19 (32)	1		
	Thatch	8/20 (40)	1		
DDT	Metal	10/25 (40)	1.46	0.41-5.24	0.56
	Thatch	1/14(7)	0.10	0.10-0.94	0.045
Bendiocarb	Metal	11/25 (44)	1.79	0.50-6.35	0.37
	Thatch	2/13 (15)	0.29	0.05-1.67	0.17
Pirimiphos methyl	Metal	9/20 (45)	2.19	0.58-8.35	0.25
	Thatch	4/19 (21)	0.34	0.08-1.43	0.14

* Adjusted for the number of sleepers in each room. Each insecticide was compared with the corresponding control roof type (metal / thatch)

### Focus Group Discussions

Spraying with any of the three insecticides was generally perceived as a necessary nuisance (Table S2). Participants disliked moving their furniture, having sprayers work unattended in their homes, waiting outside for two hours and the stains on the walls. The majority, however, said they were pleased with the spraying and were ‘rewarded with peace and health’. A few participants did not want their house sprayed, noting that ‘the mosquitoes did not die and the number of insects has slowly increased’. Most villagers did not state an intervention preference, but a few preferred bed nets: ‘[bed nets] last longer than spraying” and “can be taken outside for use’ and a few preferred IRS as 'in the hot season it is too hot to sleep under a net'. Insecticide odour was discussed frequently. The smell of DDT three days after spraying was perceived as intense but this waned and eventually disappeared: several observed that ‘The smell has gone and so mosquitoes return’. Participants from homes treated with pirimiphos methyl also noticed the smell, which ‘got less over time’ but ‘when you come close to the wall, you can smell it still now’. One villager observed: ‘because of the smell most mosquitoes are gone, but not all of them and they are coming back again’. Some residents also said that the number of rats and geckos had decreased. Many participants said that they continued to use their bed nets, for example in the in pirimiphos methyl-treated groups many slept under a net because ‘we can still hear mosquitoes’. Others in the DDT and bendiocarb groups mentioned that since IRS there were no mosquitoes they did not use the LLIN: “Even children slept without nets, as no mosquitoes were in the house. Mosquitoes are however starting to come back” and “We only bring down the net to protect us from the cold”.

## Discussion

In the study area over 50% of *An. gambiae* s.l. were resistant to both pyrethroids and DDT, although we found low or no resistance to the carbamate and the organophosphate. Twenty five years ago, shortly after permethrin-treated nets were introduced, 8.5% *An. gambiae* survived a lower discriminatory dose of permethrin for 1 hour and none survived 4% DDT, suggesting little or no resistance [23]. More recently, in 2008, just before introduction of IRS with DDT, a countrywide survey reported no resistance to either pyrethroids or DDT in samples from Basse, 15 km from Sare Alpha, but only 19-22 mosquitoes were tested for each (CI 82-100%) [24]. The current results thus indicate a rise in resistance, or at least considerable heterogeneity. A similar rise in resistance has also been reported in 2010 in Dielmo, a village in neighboring Senegal [25].

The phenotypic results from the current study site are supported by a high frequency of *kdr* point mutations in the voltage-gated sodium channel known to be associated with knockdown resistance to DDT and pyrethroid insecticides [26]. In *An. gambiae* s.s. the *kdr* 1014 leucine to phenylalanine resistant mutation approached fixation. Similar levels of kdr frequencies have been found in many areas of the sub-region [27]. The phenylalanine mutation was also present in *An. arabiensis*, but at a lower level than in *An. gambiae* s.s., and the serine mutation was found in this sibling species at a relatively high frequency compared with previous reports [3]. The rise in pyrethroid-resistance is probably due to the recent scale-up of pyrethroid-treated nets in The Gambia and Senegal, the common use of pyrethroids for agriculture in the area, the recent country-wide IRS campaigns with DDT carried out in The Gambia since 2009 and smaller scale IRS campaigns in Senegal using pyrethroids.

This study compared the use of two alternative classes of insecticide for IRS, bendiocarb and pirimiphos methyl, to which little or no resistance was found. Cone tests showed that the three insecticides persisted on mud and matt-painted walls for at least five months after spraying, with no significant differences in residual activity between insecticides in this small scale study. Similar persistence in village based studies has been previously reported for DDT and bendiocarb [8,28,29]. The micro-encapsulated form of pirimiphos methyl showed longer persistence than the two to three months previously reported for the non-encapsulated formulation [8,11,12].

The density of indoor, blood-seeking *An. gambiae* s.l. was low. IRS reduced mosquito entry in rooms with a thatch roof but no significant effect was detected on houses with metal roofs. Since thatched roofs were sprayed, but not metal ones, this may reflect the larger surface area sprayed in thatched houses and/or a greater persistence of DDT, and perhaps the other insecticides, on this surface [30].

The FGD were limited by the small-size of the study, a single village with a predominant ethnic group, and their exploratory nature. In addition, qualitative data are subject to a number of biases including partial inclinations according to the interviewees’ judgment of what the interviewers wished to hear, or by reluctance to talk about sensitive issues; these tendencies were especially true of the younger women, although rigorous measures were taken throughout to reduce such biases. In general residents indicated that IRS was a necessary nuisance and most wanted both bed nets and IRS treatment. For all treatments nearly everyone stated there were fewer mosquitoes after IRS but they increased progressively over time. Many perceived that the increase was associated with diminishing insecticide odours. In pirimiphos methyl-treated rooms, residents were of the opinion that most inhabitants were using their bed nets while for the other two treatments many mentioned that they did not sleep under a net after IRS. An important motivation for using bed nets in The Gambia is to escape the nuisance of mosquito bites [31], although high ambient temperatures discourage their use, especially towards the end of the rains [32].

One of the reasons many countries still recommend DDT for IRS is its low cost, a major factor considering that the two main components of the costs of delivering IRS are the population covered and the insecticide [33]. As both bendiocarb and pirimiphos methyl are more expensive than DDT, resistance management programmes in which insecticides are rotated will be more expensive and there is a need for relevant cost-benefit analyses.

The current study documents two nearby locations of high resistance to DDT and permethrin in The Gambia. Pyrethroids are the only insecticide available for mosquito net treatment, sleeping under treated nets in an area of high pyrethroid resistance gives similar protection to a non-treated net [34] and non-treated nets are considerably less effective against malaria [35]. These results further support the urgent and well recognised need for malaria control programmes to develop and apply effective insecticide resistance management strategies [4]. We have also demonstrated, in a rural West African village, that insecticides from the two alternative classes persist at least five months on a variety of wall surfaces and were acceptable to the population. This suggests that IRS rotations with bendiocarb and microencapsulated pirimiphos methyl are likely to be useful tools for incorporation into resistance management strategies.

## Supporting Information

Table S1 **Multivariable GEE estimates for the persistence of insecticide estimated by mosquito mortality in cone tests adjusted for wall surface, month-post IRS and insecticide.**
(DOCX)

Table S2 **All participants’ comments from the focus group discussions grouped by topic, insecticide used for IRS in the participant’s house and the gender /age of the group.**
(DOCX)
