# Supplementary material for: Alternative Treatments for Indoor Residual Spraying for Malaria Control in a Village with Pyrethroid- and DDT-Resistant Vectors in The Gambia
Source: PLoS One. 2013 Sep 13;8(9):e74351. doi: 10.1371/journal.pone.0074351 (PMC3772946; doi:10.1371/journal.pone.0074351)
Supplement: Table S1 — Multivariable GEE estimates for the persistence of insecticide estimated by mosquito mortality in cone tests adjusted for wall surface, month-post IRS and insecticide. (DOCX) [file pone.0074351.s001.docx]

Table S1

Multivariable GEE estimates for the persistence of insecticide estimated by mosquito mortality in cone tests adjusted for wall surface, month-post IRS and insecticide.

|  | Percentage mortality , ranked | | |
| --- | --- | --- | --- |
|  | Coefficient | S E. | *p* value |
| **Insecticide** |  |  |  |
| Pirimiphos methyl | . | . | . |
| Bendiocarb | -7.655 | 5.608 | 0.172 |
| DDT | -13.594 | 5.568 | 0.015 |
| **Wall surface** |  |  |  |
| Matt paint | . | . | . |
| Mud | -43.996 | 8.448 | 0.001 |
| **Time after IRS** |  |  |  |
| Month 2 | . | . | . |
| Month 3 | -16.656 | 8.209 | 0.042 |
| Month 4 | -43.989 | 8.209 | 0.001 |
| Month 5 | -45.489 | 8.209 | 0.001 |
| **Interaction of wall surface and time after IRS** |  |  |  |
| Mud and month 2 | . | . | . |
| Mud and month 3 | 49.725 | 11.528 | 0.001 |
| Mud and month 4 | 64.6 | 11.528 | 0.001 |
| Mud and month 5 | 53.767 | 11.528 | 0.001 |
