# Supplementary material for: Alternative Treatments for Indoor Residual Spraying for Malaria Control in a Village with Pyrethroid- and DDT-Resistant Vectors in The Gambia
Source: PLoS One. 2013 Sep 13;8(9):e74351. doi: 10.1371/journal.pone.0074351 (PMC3772946; doi:10.1371/journal.pone.0074351)
Supplement: Table S2 — All participants’ comments from the focus group discussions grouped by topic, insecticide used for IRS in the participant’s house and the gender /age of the group. (DOCX) [file pone.0074351.s002.docx]

| Table S2  All participants’ comments from the focus group discussions grouped by topic, insecticide used for IRS in the participant’s house and the gender /age of the group. | | | |  |
| --- | --- | --- | --- | --- |
|  |  |  |  |  |
| Key:  Group: Male, adult males; OF, older females; YF, younger females | | | |  |
| Insecticides: Pirimiphos, microencapsulated pirimiphos methyl   \| Topic \| Sub-topic \| Topic guides \| \| --- \| --- \| --- \| \| Spraying process \| 1 \| IRS team in house (alone) \| \| Spraying process \| 2 \| Preparation by householders \| \| Spraying process \| 3 \| Time taken for IRS \| \| Spraying process \| 4 \| Actions after the IRS team leave \| \| Effect of IRS on house \| 5 \| Appearance of house post-IRS \| \| Effect of IRS on house \| 6 \| Smell of house post-IRS \| \| Effect on insects & health \| 8 \| Effect of IRS on insects \| \| Effect on insects & health \| 9 \| Effect of IRS on mosquitoes \| \| Effect on insects & health \| 10 \| Effect of IRS on health \| \| Effect on insects & health \| 11 \| Other effects of IRS \| \| Effect on insects & health \| 12 \| Effect of IRS with time \| \| Sleeping under bednets \| 15 \| Participants use of nets last night \| \| Sleeping under bednets \| 16 \| Reason to use or not use their net \| \| Sleeping under bednets \| 17 \| Did children sleep under nets \| \| Concluding \| 26 \| Beneficiaries from IRS \| \| Concluding \| 27 \| Acceptance of IRS in the future \| \| Concluding \| 28 \| Preferred prevention method \| | | | |  |
|  |  |  |  |  |
| Topic: Spraying Process (sub-topics 1-4) | | | |  |
|  |  |  |  |  |
| Q_no | Insecticide | Group | Comments | sub-topic |
| 3 | DDT | Male | <<No there is no problem, is for your own good>> | 1 |
| 4 | DDT | Male | <<Yes, is a problem, because if I just come from the farm and you coming and say I should remove all my things I will be in a haste and forget something like food items inside. When spray touches it can cause problems too. Helping someone is good, but in a good way that you will not harm the person at the end too is very important.>> | 1 |
| 5 | DDT | Male | <<My house was sprayed while I was away and all my belongings were inside. I was not aware.>> | 1 |
| 6 | DDT | Male | <<They should inform us that they are coming so and so day to spray. That is something good that we should know, because a white person, many want to help, but not cause harm after the help too.>> | 1 |
| 7 | DDT | Male | <<All we see about it is peace, because all the mosquitoes and other insects will all die.>> | 1 |
| 82 | DDT | OF | <<Just come and say we have come to spray is difficult to us, but we would not want them to go without spraying.>> | 1 |
| 83 | DDT | OF | <<Is boring, if they meet you working or you are about to go to the farm.>> | 2 |
| 84 | DDT | OF | <<They should give us time and say so so date we are coming for spraying be ready.>> | 2 |
| 85 | DDT | OF | <<what we prefer is for them to inform us before coming.>> | 2 |
| 86 | DDT | OF | <<not short, but its ok, as all things are outside the house.>> | 2 |
| 87 | DDT | OF | << No is not long, because then we use to remove all the things we need out of the house.>> | 2 |
| 46 | DDT | YF | <<Yes, we trust them and the spray is for our good health.>> | 2 |
| 47 | DDT | YF | << It is better that they inform us about their coming prior to the day.>> | 2 |
| 48 | DDT | YF | << for us whatever is good is ok.>> | 2 |
| 49 | DDT | YF | <<No, as it is for helping us.>> | 2 |
| 50 | DDT | YF | <<We have just removed our things and sit outside until they finish.>> | 2 |
| 134 | bendiocarb | Male | <<There is no problem in that they just came to help us. There is no distrust as we take out our things.>> | 2 |
| 135 | bendiocarb | Male | <<You know there is nothing you can tell the person, just bear the person, because the person did not come to spoil you, they have come to help. You just wait for the person to finish you wait some then you bring in your things. You are rewarded with peace and health.>> | 2 |
| 136 | bendiocarb | Male | << Just stop the work until the time is up for you, then you go in and take in your things.>> | 2 |
| 229 | bendiocarb | OF | <<There is no problem in that we are the ones they are helping. >> | 2 |
| 230 | bendiocarb | OF | <<No suffering in it because we are the ones they came to help. The spray is very good.>> | 2 |
| 231 | bendiocarb | OF | <<No, it does not disturb us, because we use to remove all we need from the house.>> | 2 |
| 182 | bendiocarb | YF | <<Because me I was cooking lunch when they just came to my house and say remove all your things. We want to pomp the house.>> | 2 |
| 183 | bendiocarb | YF | << Is difficult when coming from farms as we are tired. But for our own interest.>> | 2 |
| 184 | bendiocarb | YF | <<When visitor is present it would be a problem and prefer spraying was done the next day.>> | 2 |
| 185 | bendiocarb | YF | <<Some people will feel angry and they may not feel like removing out their things. But they should not do that, because it is for their own benefit. People are not the same. If the person does not want his or her house to be sprayed, they don’t force you, because it is not by force. They don’t force you because it is for your own betterment.>> | 2 |
| 186 | bendiocarb | YF | <<For us all they have done is good, it is for our own health, because when they ask us to remove all our things and spray our houses, this is for our own good. It has helped us, really. Because even outside there are no mosquitoes.>> | 2 |
| 187 | bendiocarb | YF | << If there is at all a program in our house, we cannot take all our things outside. Then we can ask them to go until some time. You, the compound head, and your family cannot be outside or ask your visitors to stay outside at that time. Then we say: wait until next time.>> | 2 |
| 188 | bendiocarb | YF | << ( I was planning to go to the farm)I stop going to the farm and wait for our house to be sprayed. But we would have preferred we are informed two or three days before spraying.>> | 2 |
| 191 | bendiocarb | YF | << It’s difficult, but if at all they brought money and you have to wait for some time outside before you can go in and then they will give you the money. You will have patience, so for that being the case, it’s all the same. Because this is not money, but for your own health.>> | 2 |
| 194 | bendiocarb | YF | <<After two hours they sweep all the house and they scrub it until it’s clean.>> | 2 |
| 195 | bendiocarb | YF | <<All the insects died on the floor, we had to clean it. >> | 3 |
| 272 | primiphos | Male | <<We did not see anything missing from the house and the spray has its benefits.>> | 3 |
| 273 | primiphos | Male | << For me, I have no problem with it, because when they came to our compound they asked the women to move out all materials.>> | 3 |
| 274 | primiphos | Male | <<… it would have been better if the villagers were included in the spraying. So that they can inform the people prior to the spraying date, better than coming down from a vehicle and telling us to remove our things from our houses.>> | 3 |
| 275 | primiphos | Male | << no, because it is for our own benefit and health>> | 3 |
| 276 | primiphos | Male | << We sat outside till it was time to get inside and put back our things>> | 3 |
| 345 | primiphos | OF | <<That is very good, we did not see any problem in that except our own benefit.>> | 3 |
| 346 | primiphos | OF | <<They are all the time after our own good, if you see you are going out of your house and leaving only one person in your house it is because of your own good and also the trust you have in them. >> | 3 |
| 348 | primiphos | OF | << Some agree, some will say no, we don’t have time till next time>> | 3 |
| 349 | primiphos | OF | <<they should tell us that today we are coming.>> | 3 |
| 350 | primiphos | OF | <<The time they came to us, it was raining and I was outside with all our things under the rain. >> | 3 |
| 351 | primiphos | OF | <<The time they were spraying it was not cloudy, but it was in my absence and they call my children and asked them to remove all things outside. Then I told my children that if the houses are sprayed, let them stay outside until the smell goes off. But then it was raining.>> | 3 |
| 352 | primiphos | OF | <<No nobody enters the house.>> | 3 |
| 320 | primiphos | YF | <<Yes, Is for your own good, but sometimes ... there are some of your things that if you are asked to take out you may not like it, because there are things you may not want people to see outside. You will not be happy.>> | 3 |
| 321 | primiphos | YF | <<There is no difficulty in when you are asked to remove all your belongings. It will not do anything to you.>> | 4 |
| 322 | primiphos | YF | << It is difficult and is also boring because if you are asked not to enter your own house for like two hours and you need things from your house or maybe you need money to cook your lunch it will definitely make your whole work later.>> | 4 |
|  |  |  |  |  |
| Topic: Effect of IRS on house (sub-topics 5,6) | | | |  |
|  |  |  |  |  |
| no | Insecticide | Group | Comments | Sub-topic |
| 8 | DDT | Male | <<It look like matt paint on it. It is white.>> | 5 |
| 9 | DDT | Male | <<it takes up to 2 or 3 days, that if you don’t burn churrai, you cannot cope with the smell.>> | 6 |
| 10 | DDT | Male | <<For me when it was sprayed that night I don’t burn any churai and nothing happened to me.>> | 6 |
| 88 | DDT | OF | <<dead insects and the smell of the spray, we smell a lot and lines of spray on the wall just like matt paint.>> | 5 |
| 90 | DDT | OF | <<smell is still present. It stays, even now you can smell it.>> | 6 |
| 51 | DDT | YF | <<There is a white colour on the walls and some particles on the floor with odour.>> | 5 |
| 52 | DDT | YF | <<It left a whitish colour on the walls and some strong odour.>> | 5 |
| 137 | bendiocarb | Male | "6/7 stains, although one said this was "small bit" | 5 |
| 140 | bendiocarb | Male | <,Don’t mind stains as it is for our own health.>> | 5 |
| 141 | bendiocarb | Male | <<For me it doesn’t do anything to me unless makes the walls white.>> | 5 |
| 142 | bendiocarb | Male | <<Smell of the spray, the gas remains there and all the small insects fall on the ground.>> | 5 |
| 143 | bendiocarb | Male | <<No, there is no smell now anywhere.>> | 6 |
| 232 | bendiocarb | OF | " 4/6 said they saw stains on the walls" | 5 |
| 234 | bendiocarb | OF | <<It looks like matt paint is applied. It smells.>> | 5 |
| 235 | bendiocarb | OF | <<Smell was present for the first four days.>> | 6 |
| 236 | bendiocarb | OF | << No, it does not cause anything to us. >> | 6 |
| 196 | bendiocarb | YF | <<The walls of my house look like we applied matt paint. >> | 5 |
| 198 | bendiocarb | YF | <<The house used to smell with the spray, …. we burn churai until the smell goes out.>> | 6 |
| 199 | bendiocarb | YF | <<Still now we have help from the sprayers then whatever smell it takes, it still smells but we will bear it, it is for our health>> | 6 |
| 200 | bendiocarb | YF | << My house was sprayed and I slept in the house that night, but nothing was wrong with me. It was only good. We discovered something good, but not something bad. >> | 6 |
| 277 | pirimiphos | Male | <<There is an odour and the walls do have a whitish colour.>> | 5 |
| 278 | pirimiphos | Male | <<It appears white.>> | 5 |
| 279 | pirimiphos | Male | <<A lot of (white lines), but the lines to disappear after some time. The spray has benefits and we want it to be twice each year, at the beginning of the rainy season and in the middle.>> | 5 |
| 280 | pirimiphos | Male | <<For me, after spraying there were some white lines and up to three weeks there was an odour. But all was to help me>> | 5 |
| 281 | pirimiphos | Male | <<Just for a few days, but as for the benefits, they stay.>> | 6 |
| 282 | pirimiphos | Male | << This odour does not disturb us as it is not very strong.>> | 6 |
| 283 | pirimiphos | Male | <<no smell after two days.>> | 6 |
| 284 | pirimiphos | Male | <<When you come close to the wall you can smell it still now, but in general smell decreases.>> | 6 |
| 356 | pirimiphos | OF | <<stains which look like matt paint.>> | 5 |
| 357 | pirimiphos | OF | << It used to be white with line coming down to the floor of the house. >> | 5 |
| 358 | pirimiphos | OF | <<Stains even on the floor.>> | 5 |
| 359 | pirimiphos | OF | << The spray used to smell about two days. We have to burn churai. >> | 6 |
| 323 | pirimiphos | YF | <<It does not have any smell, but the walls used to be white and lines towards the floor.>> | 5 |
| 324 | pirimiphos | YF | <<Yes there are white lines down to the floor.>> | 5 |
| 325 | pirimiphos | YF | <<When it is sprayed it does (smell) we have to burn churai to do away with the smell those days, but it got less over time>> | 6 |
| 326 | pirimiphos | YF | << For me the day the house was sprayed I forget to remove my bed sheet. Then late in the night my husband wakes me up and says I should remove the bed sheet because he was having headache from the smell of the bed sheet. >> | 6 |
|  |  |  |  |  |
| Topic: The effects of IRS on insects and health (sub-topics 8-12) | | | |  |
|  |  |  |  |  |
| Qno | Insecticide | Group | Comments | Sub-topic |
| 11 | DDT | Male | <<The number of mosquitoes has dropped, but cases of malaria have increased.>> | 8 |
| 12 | DDT | Male | <<(the people of the house benefit) because mosquitoes will not enter the house that is sprayed.>> | 8 |
| 13 | DDT | Male | <<For me still now, the mosquitoes did not die and the number of insects has slowly increased in my house. I don’t know whether it is because my house is at the last part of the village.>> | 8 |
| 14 | DDT | Male | <<No I did not see anything (dead mosquitoes/ insects) that day, but after three days I have to sleep under a net or burn mosquito coil.>> | 9 |
| 15 | DDT | Male | << Not all of them (mosquitoes) died, some are left still.>> | 9 |
| 16 | DDT | Male | <<The previous spray used increased the mosquito population but this one has decreased their population.>> | 9 |
| 17 | DDT | Male | <<Still now the houses are been sprayed and nets are given, but this year malaria cases increase more. Kids die after only two days of being sick. >> | 10 |
| 91 | DDT | OF | << It kills all insects including mosquitoes in house.>> | 8 |
| 93 | DDT | OF | <<It is good, because it protects us from lots of diseases.>> | 10 |
| 53 | DDT | YF | <<Even at night if you return home, there is a big difference between sprayed compounds and not sprayed.>> | 8 |
| 54 | DDT | YF | <<It’s good, it will increase our health and mosquitoes will not bite.>> | 9 |
| 55 | DDT | YF | <<…as the spray kills even the small insects, if these get into someone’s ears, that person has to go to the health facility. >> | 9 |
| 56 | DDT | YF | <<Because the way the mosquitoes disturb you is different when the house is sprayed. We can still hear mosquitoes behind the net.>> | 9 |
| 57 | DDT | YF | <<It’s good, will increase our health and mosquitoes will not bite.>> | 10 |
| 59 | DDT | YF | <<(If the spraying stopped then) the mosquitoes and other insects will return.>> | 12 |
| 147 | bendiocarb | Male | <<Does not disturb our health.>> | 10 |
| 149 | bendiocarb | Male | <<Yes, they come back small small (with time).>> | 11 |
| 237 | bendiocarb | OF | <<(after sometime, the insects) come back small small, but you should spray again.>> | 8 |
| 238 | bendiocarb | OF | <<There used to be no mosquitoes and the spray even used to kill insects and even mice, because of the smell. The smell used to stay for about two to three days. The spray used to kill even insects that spoiled wood. The smell has gone and so the mosquitoes return>> | 8 |
| 241 | bendiocarb | OF | << Is all about peace and peace because if sweep our houses and burn churrai we are free of biting from mosquitoes.>> | 10 |
| 201 | bendiocarb | YF | << yes, nowadays there are no mosquitoes, but whenever they comes we want help from you.>> | 7 |
| 203 | bendiocarb | YF | <<We have no mosquitoes in our compounds and also we are safe from malaria. The spraying had helped us a lot, we will not have malaria. >> | 8 |
| 205 | bendiocarb | YF | <<all small insects and mosquitoes and rats, all of them dies quickly.>> | 9 |
| 206 | bendiocarb | YF | <<…. health increases because if all the mosquitoes in your house die, then your health increases. Then you don’t have to be going to the hospital every time. If it is not something from God, nothing will happen to you.>> | 10 |
| 207 | bendiocarb | YF | << Last year we are not able to sleep, because of mosquitoes, but this year we sleep without bringing down our nets or curtains in our bedrooms.>> | 10 |
| 208 | bendiocarb | YF | <<no health problems. Now better as insects in grass of roof don't crawl into our ears. Saves us a lot of trouble like going to the hospital.>> | 10 |
| 224 | bendiocarb | YF | <<Still now we have help from the sprayers then whatever smell it takes, it still smells but we will bare it, it is for our own health. >> | 12 |
| 285 | pirimiphos | Male | <<The spray removes two things from the house: mosquitoes and other small insects>> | 9 |
| 287 | pirimiphos | Male | <<It is good and brings development,>> | 10 |
| 289 | pirimiphos | Male | <<… the spray has helped, as the coming of the mosquitoes has been delayed up to the time of harvesting; still we are seeing the benefit>> | 12 |
| 360 | pirimiphos | OF | <<because it kills the insects in our house>>. | 8 |
| 361 | pirimiphos | OF | <<It kills small insects and mosquitoes and also gecko and mice.>> | 8 |
| 363 | pirimiphos | OF | << it will be a health for the person and also benefit, because no insects will drop on your food anymore.>> | 10 |
| 365 | pirimiphos | OF | << It will free us from biting.>> | 10 |
| 366 | pirimiphos | OF | <<Also will protect us from being sick and having someone sick as well.>> | 10 |
| 367 | pirimiphos | OF | <<Now (3 months after IRS) they (the mosquitoes) have reduced, but not totally.>> | 12 |
| 369 | pirimiphos | OF | <<This year the mosquitoes reduce, but are coming back small small again. Because of the smell most mosquitoes are gone, but not all of them and they are coming back again. >> | 12 |
| 328 | pirimiphos | YF | <<The spray kills all insects in the house.>> | 8 |
| 329 | pirimiphos | YF | <<In some places all the insects and mosquitoes will die after the spray.>> | 9 |
| 330 | pirimiphos | YF | <<All the insects die including all the rats because of the smell in the house.>> | 11 |
|  |  |  |  |  |
| Topic: Would they like their houses to be sprayed again? (27) | | | |  |
|  |  |  |  |  |
| Q_no | Insecticide | Group | Comments | sub-topic |
| 40 | DDT | Male | << For me, even today if I am asked I will say I want it again. It is for my own benefit.>> | 27 |
| 121 | DDT | OF | << We will not say no and those that did not have the spraying should have it too. As it kills all the insects and mosquitoes and protects us from being sick and our people being sick.>> | 27 |
| 73 | DDT | YF | <<Yes, as we have seen the benefits.>> | 27 |
| 74 | DDT | YF | <<Yes>> | 27 |
| 172 | bendiocarb | Male | << Yes, we will agree, because you cannot say no to something that is good for you. | 27 |
| 263 | bendiocarb | OF | <<Yes we will spray and all others will spray too.>> | 27 |
| 265 | bendiocarb | OF | <<We all want houses to be sprayed.>> | 27 |
| 311 | pirimiphos | Male | <<Yes, but all compounds and not just a few.>> | 27 |
| 390 | pirimiphos | OF | << For me, if the spray comes back I will say I am ok, because my house was sprayed, but still there are mosquitoes.>> | 27 |
|  |  |  |  |  |
| Topic: Use of LLIN by adults and children (sub-topics 15-17) | | | |  |
|  |  |  |  |  |
| Qno | Insecticide | Group | Comments | Sub-topic |
| 28 | DDT | Male | <<More people do not sleep under a net than the people that do. A lot of people don’t use nets nowadays. We only bring down the net to protect us from the cold. >> | 15 |
| 30 | DDT | Male | <<I, (name), is because there are no mosquitoes in my house. Before I used nets but now because of the cool, I don’t. >> | 16 |
| 31 | DDT | Male | <<Is because still there are some small mosquitoes around and our compound is at the end of the village.>> | 16 |
| 32 | DDT | Male | <<outside they use nets, but inside they don’t. >> | 17 |
| 33 | DDT | Male | <<The nets also are useful in preventing or reducing cold at night, therefore they are still useful for the children.>> | 17 |
| 106 | DDT | OF | <<For me, outside we don’t use nets because it used to be windy and there used to be no mosquitoes, but inside we do use nets.>> | 15 |
| 107 | DDT | OF | <<We slept under a net.>> | 15 |
| 108 | DDT | OF | <<For me I came from the village I was very tired I could not bring down my net so I just slept like that.>> | 15 |
| 109 | DDT | OF | <<Because we are used to sleeping under nets.>> | 16 |
| 110 | DDT | OF | <<Because still now there are mosquitoes.>> | 16 |
| 111 | DDT | OF | <<To protect ourselves from biting.>> | 16 |
| 112 | DDT | OF | <<Yes, they (children) are used to sleep under nets.>> | 17 |
| 62 | DDT | YF | <<Outside we sleep in nets, but not inside the houses. >> | 15 |
| 64 | DDT | YF | << Some do (children sleep under nets), others don’t. There are no mosquitoes>> | 17 |
| 155 | bendiocarb | Male | <<We sleep under net as it protects just in case mosquitoes come in.>> | 15 |
| 156 | bendiocarb | Male | <<For me, we do not sleep under net even children sleep without nets, as no mosquitoes were in the house. Mosquitoes however are starting to come back.>> | 15 |
| 159 | bendiocarb | Male | <<(doesn't sleep under net) because the houses are sprayed and there are no mosquitoes. But they will bite outside.>> | 16 |
| 161 | bendiocarb | Male | << They (women) don’t use nets outside. They just cover their children with a wrap and say it is cool now so there are no mosquitoes.>> | 17 |
| 162 | bendiocarb | Male | <<Not all of them (children), some use nets but some don’t.>> | 17 |
| 249 | bendiocarb | OF | << Is because the mosquitoes have reduced, it is cold now. >> | 16 |
| 250 | bendiocarb | OF | << No mosquitoes>> | 16 |
| 253 | bendiocarb | OF | << Yes,(the children use nets) when sleeping outside.>> | 17 |
| 213 | bendiocarb | YF | <<when we sleep outside, we sleep under bednet. But when we sleep in our houses we do not use bednets, as there are no mosquitoes.>> | 15 |
| 216 | bendiocarb | YF | <<It is to protect ourselves from mosquitoes when we sleep under bednets then we will not have malaria disease.>> | 16 |
| 215 | bendiocarb | YF | <<All the houses in our compound have nets. Even the house of our boy children has nets.>> | 17 |
| 295 | pirimiphos | Male | << It’s a long time since I last slept under a net.>> | 15 |
| 296 | pirimiphos | Male | <<Me, I have no net.>> | 15 |
| 297 | pirimiphos | Male | <<I got a net, but since my house was sprayed, I did not use it.>> | 15 |
| 299 | pirimiphos | Male | <<After spraying there are no mosquitoes, but I still bring down the net.>> | 15 |
| 300 | pirimiphos | Male | <<Yes they (children) sleep with their mothers under a net.>> | 17 |
| 301 | pirimiphos | Male | <<I sleep with my children and we don’t have a net.>> | 17 |
| 375 | pirimiphos | OF | <<We sleep under net because that is their use.>> | 15 |
| 376 | pirimiphos | OF | <<I don’t use it because no mosquitoes are biting me.>> | 15 |
| 377 | pirimiphos | OF | <<We use nets, we heard mosquitoes making noise outside the net. >> | 15 |
| 378 | pirimiphos | OF | <<No mosquitoes are biting me.>> | 16 |
| 379 | pirimiphos | OF | <<(Mosquitoes) reduced more this year, so some people just don’t want to use nets or sleep under them.>> | 16 |
| 380 | pirimiphos | OF | <<We heard mosquitoes making noise outside the net. >> | 16 |
| 381 | pirimiphos | OF | <<Yes, we using nets for our children.>> | 17 |
| 335 | pirimiphos | YF | <<For me I did not sleep under a net, because since after the spray there are no mosquitoes in the house.>> | 16 |
| 336 | pirimiphos | YF | <<For me in my house even if the children are outside they use bednets.>> | 17 |
| 337 | pirimiphos | YF | <<In our compound there is one woman who doesn’t sleep under a net and doesn’t let her children do so as well.>> | 17 |
|  |  |  |  |  |
| Topic: Preferred prevention method, nets or IRS? (28) | | | |  |
|  |  |  |  |  |
| Qno | Insecticide | Group | Comments | Sub-topic |
| 41 | DDT | Male | <<We would take nets because the spray would not last for long time like one year, but the nets last longer than spraying.>> | 28 |
| 75 | DDT | YF | <<nets and spraying, as it works better.>> | 28 |
| 76 | DDT | YF | <<Combination is better as the spray drives away the insects and gecko’s and rats.>> | 28 |
| 77 | DDT | YF | <<Nets protect even if not everything is killed from spraying. They won’t reach you.>> | 28 |
| 127 | DDT | OF | <<We want both spray and nets, because spray as time goes on will not be effective, but the nets can protect you after that. Therefore if we have to choose, we choose nets, because spraying is only useful in the house, but nets can be taken outside and hung it.>> | 28 |
| 173 | bendiocarb | Male | <<Whatever is better. The nets are the best.>> | 28 |
| 174 | bendiocarb | Male | <<some will say they want spray, because in the hot season it is too hot sleep under nets.>> | 28 |
| 175 | bendiocarb | Male | <<Nets makes one itch, so he prefers spraying.>> | 28 |
| 266 | bendiocarb | OF | <<We want nets with spray.>> | 28 |
| 267 | bendiocarb | OF | <<You want rice and sauce, not just rice or just sauce.>> | 28 |
| 312 | pirimiphos | Male | <<Both nets and spraying as each have a separate role.>> | 28 |
| 313 | pirimiphos | Male | <<Spraying increases cleaning (more insects on the floor).>> | 28 |
| 314 | pirimiphos | Male | <<Nets make the bed more civilized.>> | 28 |
| 342 | pirimiphos | YF | <<We want spraying together with nets.>> | 28 |
| 391 | pirimiphos | OF | <<We prefer nets.>> | 28 |
| 392 | pirimiphos | OF | <<We want nets together with spraying.>> | 28 |
